# Supplementary material for: Correlation Analysis of Microbial Contamination and Alkaline Phosphatase Activity in Raw Milk and Dairy Products
Source: Int J Environ Res Public Health. 2023 Jan 19;20(3):1825. doi: 10.3390/ijerph20031825 (PMC9915017; doi:10.3390/ijerph20031825)
Supplement: Supplementary file 1 [file ijerph-20-01825-s001.zip › ijerph-2063982-supplementary.pdf]

Supplementary Table S1. Sampling amount for each province.

**Province (municipality)**	**Sampling Amount**
Hebei	106
Neimenggu	110
Heilongjiang	120
Shanghai	103
Jiangsu	111
Zhejiang	126
Fujian	111
Shandong	143
Henan	112
Hubei	101
Guangdong	112
Shanxi	100
Gansu	148
Total amount	1503
